# Emerging Insights into the Role of Trace Elements in Human Health and Disease Prevention

**DOI:** 10.3390/nu17233678

**Published:** 2025-11-25

**Authors:** Qingsong Li, Xuefeng Yu, Haobiao Liu, Liangjia Wang, Jing Han

**Affiliations:** School of Public Health, Health Science Center, Xi’an Jiaotong University, Xi’an 710061, China; 17380784728@163.com (Q.L.); 13685537748@163.com (X.Y.); houbiulau@gmail.com (H.L.); wangliangjia_1005@163.com (L.W.)

Trace elements, though required only in minute quantities, are indispensable for the proper functioning of biological systems. They serve as cofactors for numerous enzymes, maintain redox equilibrium, and play pivotal roles in cellular signaling, immune modulation, and hormonal regulation. Both deficiency and excess can disturb physiological homeostasis, leading to a wide spectrum of metabolic, neurological, reproductive, and musculoskeletal disorders. Recent advances have further underscored the essential contribution of trace elements to the pathogenesis and prevention of complex diseases.

This Special Issue, “*A New Perspective: The Effect of Trace Elements on Human Health*,” brings together seven high-quality contributions that collectively illuminate the biological, clinical, and public health relevance of trace element balance. Spanning diverse physiological systems—from hepatic metabolism and reproductive health to cognition, endocrinology, and musculoskeletal integrity—these studies reveal how elemental regulation intersects with oxidative stress, inflammation, and endocrine signaling. Together, they reinforce a growing scientific consensus: maintaining trace element homeostasis is fundamental to sustaining human health.

Manganese (Mn) is an essential cofactor for enzymes such as mitochondrial superoxide dismutase, which protects cells from oxidative damage [1,2]. Two complementary studies in this Special Issue explore the systemic consequences of Mn deficiency. Dr. Hu et al. provided a comprehensive analysis of how Mn deprivation reshapes hepatic physiology (contribution 1). Through transcriptomic and biochemical profiling, the authors demonstrated that Mn deficiency amplifies hepatic oxidative stress, impairs lipid metabolism, and activates proinflammatory signaling pathways. These perturbations ultimately disrupt energy balance and induce liver injury, highlighting the liver’s sensitivity to micronutrient imbalance. Extending these findings to reproductive physiology, Dr. Peng et al. reported that Mn deficiency caused testicular structural abnormalities, elevated oxidative biomarkers, and downregulation of the Nrf2 antioxidant pathway (contribution 2). Damage to the blood–testis barrier consequently impaired spermatogenesis and reduced sperm quality. Together, these studies reveal that Mn deficiency induces a multi-organ oxidative stress phenotype, positioning Mn as a key regulator of both metabolic and reproductive health.

Environmental heavy metal exposure poses an increasing threat to global health. Dr. Yang’s team contributed valuable epidemiological evidence from a longitudinal cohort study, demonstrating that higher intakes of essential minerals—including selenium, zinc, calcium, and magnesium—were linked to a reduced risk of cadmium-induced cognitive decline (contribution 3). They proposed that adequate mineral nutrition mitigates neurotoxic effects via antioxidant defense and competitive inhibition of cadmium absorption. This concept of nutritional antagonism—where protective elements buffer against toxic exposures—holds profound implications for public health nutrition, particularly in aging populations and polluted environments.

Selenium (Se), a vital component of glutathione peroxidase and other selenoproteins, contributes significantly to antioxidant defense during intense physical activity. In their study, Dr. Toro-Román et al. observed that both male and female athletes exhibited fluctuations in plasma Se concentrations, but with distinct sex-specific patterns (contribution 4). Male players showed greater Se depletion during high-intensity phases, while females maintained relative stability, possibly reflecting hormonal modulation or differences in training load. These results highlight the importance of individualized nutritional monitoring in sports medicine—considering biological sex, metabolic rate, and oxidative stress burden. Selenium status, in particular, may influence recovery capacity, inflammation control, and overall athletic performance.

In a cross-sectional investigation, Dr. Tanaka et al. explored the underrecognized relationship between trace element profiles and androgen physiology (contribution 5). Using clustering analysis, they identified distinct metabolic signatures characterized by variations in zinc, copper, and selenium concentrations, which corresponded to serum testosterone levels and related symptoms. Zinc—known to modulate 5α-reductase activity and testosterone synthesis—emerged as a critical determinant of endocrine balance. Conversely, elevated copper levels correlated with reduced testosterone and fatigue-like symptoms. This study suggests that trace element interactions may contribute to subclinical hypogonadism, even among healthy young adults, bridging nutritional science with endocrinology and lifestyle medicine.

Accurate detection of trace element imbalance is the first step toward effective prevention and intervention [3]. In a comprehensive review, Dr. López-Alonso examined the global burden of trace element deficiency and excess, highlighting limitations of conventional biomarkers—such as serum concentrations—that may not reflect intracellular or functional status (contribution 6). The authors advocated for standardized analytical protocols, multi-element profiling, and integration with omics technologies to better capture the complexity of elemental dynamics in health and disease.

Complementing this perspective, Dr. Han and colleagues reviewed the intricate relationships between trace elements and joint degeneration (contribution 7). They emphasized how imbalances in zinc, copper, and selenium disrupt chondrocyte homeostasis, promote oxidative stress, and intensify inflammatory cascades in osteoarthritis. Notably, they proposed that nutritional modulation of trace element intake could serve as an adjunct therapeutic strategy to slow cartilage degradation and improve joint function. Collectively, these reviews reflect a paradigm shift: trace element research has evolved from a narrow focus on deficiency syndromes to an integral component of precision medicine, disease prevention, and health policy formulation.

The studies featured in this Special Issue converge on a unifying insight: trace elements are not peripheral participants but central regulators of human physiology. Their intricate interactions with oxidative stress, immune regulation, and endocrine function reveal an elemental layer underlying nearly all biological processes. Yet, several critical knowledge gaps remain. First, dose–response thresholds for optimal versus toxic exposures are still poorly defined, particularly for multi-element interactions [4]. Second, individual variability—driven by genetics, sex, diet, and environmental exposure—complicates the establishment of universal reference standards [5]. Third, much of the current evidence is derived from cross-sectional or animal studies, limiting causal inference. Addressing these challenges will require integrative frameworks that combine omics technologies, longitudinal cohorts, and controlled intervention trials to elucidate causal pathways. Moreover, adopting a system’s biology perspective is essential to understand how dietary, environmental, and genetic factors converge on elemental metabolism.

Developing personalized nutritional guidelines and monitoring tools may enable targeted interventions that restore elemental balance before disease onset. Ultimately, ensuring optimal trace element status throughout the lifespan represents a cost-effective and sustainable strategy for enhancing population health. The research compiled in this Special Issue not only advances fundamental understanding but also paves the way for translating elemental biology into preventive medicine and clinical nutrition.
